# A systematic review and meta-analysis of the sensitivity of antibody tests for the laboratory confirmation of COVID-19

**DOI:** 10.2217/fvl-2021-0211

**Published:** 2021-12-15

**Authors:** Nigel A Makoah, Thomas Tipih, Matefo M Litabe, Mareza Brink, Joseph B Sempa, Dominique Goedhals, Felicity J Burt

**Affiliations:** ^1^Division of Virology, Faculty of Health Sciences, University of The Free State, Bloemfontein, 9301, South Africa; ^2^Division of Virology, National Health Laboratory Service, Bloemfontein, 9301, South Africa; ^3^Free State Department of Health, Bloemfontein, 9301, South Africa; ^4^Department of Biostatistics, Faculty of Health Sciences, University of The Free State, Bloemfontein, 9301, South Africa; ^5^DST-NRF Centre of Excellence in Epidemiological Modelling & Analysis (SACEMA), Stellenbosch University, Stellenbosch, 7600, South Africa

**Keywords:** COVID-19, laboratory diagnosis, RT-PCR, serology

## Abstract

**Aim:** The aim of this study was to investigate the utility of serological tests for the diagnosis of COVID-19 during the first week of symptom onset in patients confirmed with the real-time RT-PCR. **Materials & methods:** A systematic review and meta-analysis of 58 publications were performed using data obtained from Academic Search Ultimate, Africa-wide, Scopus, Web of Science and MEDLINE. **Results:** We found that the highest pooled sensitivities were obtained with ELISA IgM-IgG and chemiluminescence immunoassay IgM tests. **Conclusion:** Serological tests have low sensitivity within the first week of symptom onset and cannot replace nucleic acid amplification tests. However, serological assays can be used to support nucleic acid amplification tests.

COVID-19 is a pandemic caused by SARS-CoV-2. The virus first emerged in Wuhan, China in December 2019 [1] and has since spread throughout the world. Despite the availability of vaccines, infections and fatalities continue to surge globally, especially in low- and middle-income countries where vaccine rollout is currently inadequate. The emergence of SARS-CoV-2 variants with increased transmissibility rates threatens to reverse some of the gains achieved in slowing down infections. It therefore remains necessary to maintain and intensify the various control measures previously implemented to curb the viral spread.

SARS-CoV-2 is classified in the Coronaviridae family and the *Betacoronavirus* genus. The virus possesses a single-stranded positive-sense RNA genome of approximately 30 kb [2]. The genome encodes nonstructural proteins including NSP1 to NSP10 and NSP12 to NSP16, structural proteins (envelope, membrane, nucleocapsid and spike protein) and accessory proteins namely 3a, 6, 7a, 7b, 8 and 10 [2]. The structural proteins perform roles in transcription, assembly, budding, envelope formation and viral pathogenesis while the nonstructural proteins and accessory proteins are involved in viral replication [3]. Molecular diagnostic assays amplify partial spike and/or nucleocapsid genes, and serological assays target antibody detection against either the spike glycoprotein or nucleocapsid.

It is paramount to accurately diagnose COVID-19 and identify cases early enough to limit the spread. The WHO recommends using nucleic acid amplification test (NAAT), including real-time RT-PCR (rRT-PCR), as the primary testing method. The test is accurate, and hence it remains the gold standard for COVID-19 diagnosis [4].

COVID-19 is unprecedented; thus, laboratory diagnosis has been a challenge globally. Low-and middle-income countries, the majority of which are in Africa, are not exempt from the challenges as they struggle to perform large-scale diagnostic testing of COVID-19. In Africa, the challenges presented by expensive COVID-19 RT-PCR kits, limited adequately equipped laboratory facilities and the need for clinical laboratory scientists are blunting effective response to the pandemic. Consequently, underdiagnosis is resulting in undetected viral transmission making Africa a fertile ground for the emergence of SARS-CoV-2 variants. If diagnostic testing capacity is to be enhanced by using serological assays, then the limitations of serology, specifically demonstration of early antibody in the diagnostic laboratory, need to be defined for accurate interpretation of results.

Serological assays are reliable, simple, and cost-effective techniques that allow direct and indirect detection of infections [5]. Laboratory-based serological methods, such as ELISA, chemiluminescence immunoassay (CLIA) and lateral flow immunochromatographic assay (LFIA) are used as supportive diagnostic tools in the attempt of widening access to diagnosis, screening of asymptomatic persons, and providing information on the immune status of recovered persons to end isolation [6].

Serological tests generally detect IgA, IgG, IgM or total antibodies from patient sera or plasma, that are directed against the SARS-CoV-2 specific spike protein (S) and/or nucleocapsid protein (N). The kinetics of IgA, IgM and IgG antibodies against the specific SARS-CoV-2 proteins informs host immune response and is a critical application of diagnostic tests. Antibody profiling in COVID-19 patients has been described in several studies and it has been shown that an IgM antibody response is detectable as early as 3 days post illness onset and peak levels were observed between the second and the third week, while IgG antibody was detected from day four of illness with peak levels observed between the third to the fourth week [7–10]. However, after profiling IgM and IgG antibodies in 26 COVID-19 patients in one study, the immunoglobulins either appeared at the same time or varied whether IgM or IgG was detected first [8]. In yet another study, longitudinal profiling of serum, saliva and bronchoalveolar lavage fluid for SARS-CoV-2 specific antibodies, demonstrated the dominance of IgA isotypes within the first week of symptom onset [11]. These observations may highlight the importance of assaying all the three isotypes (IgA, IgM and IgG) in diagnosis of acute COVID-19. Evidence from follow-up studies of COVID-19 patients suggests that serum IgA and IgM antibodies gradually decline after achieving peak levels; while on the contrary IgG persist longer [12,13]. Current data suggest that serological testing can detect infection a few days after the onset of symptoms and might be a suitable approach to complement molecular tests and increase the diagnostic reliability. Additionally, serological tests might be instrumental in low-income countries where access to molecular testing can be difficult.

Previous systematic reviews pooled sensitivity stratified by test type and immunoglobulin class and reported lower sensitivities with the LFIA tests compared with ELISA and CLIA. Hence the use of LFIA tests in the diagnosis of COVID-19 has been questioned. The sensitivity of LFIA, CLIA and ELISA have been reported the lowest during the first week of symptom onset but peaked in the third week or later [14,15]. Even though the current evidence points to an increase in sensitivity later during infection, serological tests still result in many false negatives [14]. The use of serological tests in medical decision making should be accompanied with caution [14]. It is worthy noting that previous reviews included studies mostly from China.

In this systematic review we investigated serological assays and analyzed the results to determine the sensitivity of various serological assays for early detection of antibody response during acute phase of illness to support molecular diagnosis. Our study provides pooled sensitivity stratified by test type, immunoglobulin class and test antigen from studies across the world which included at least 300 samples to assess the performance of serological tests within 7 days of symptom onset.

## Materials & methods

### Data sources

We performed a search on 15 April 2021, using the following databases with no restriction in languages: MEDLINE, Academic Search Ultimate, Africa-Wide Information through EBSCOhost, Web of Science and Scopus. Our search terms were (antibody test or IgG or IgM or IgA) and (diagnostic or RT-PCR) and (SARS-CoV-2 or COVID-19 or coronavirus).

### Inclusion & exclusion criteria

Eligible studies were full research articles assessing serological assays as diagnostic tools for the laboratory confirmation of COVID-19. We included studies in which sensitivity and specificity of COVID-19 serological diagnostic were evaluated against the gold standard RT-PCR as reference. We excluded case reports, review articles, editorials and viewpoints. Research articles with less than 300 samples used to estimate sensitivity and specificity were excluded based on recommendations by Bujang and Adnan [16]. Three investigators (NA Makoah, M Brink and T Tipih) independently screened titles and abstracts, and three (NA Makoah, MM Litabe and M Brink) independently screened full-text papers, and disagreements were resolved by two agreeing.

### Data analysis

Four investigators (NA Makoah, T Tipih, M Brink and MM Litabe) extracted and verified data on sensitivity, specificity, serological methods, the immunoglobulin class detected and the antigens targeted.

CMIA, eCLIA and CLIA were all summarized as CLIA because of the similarities in the method.

### Statistical analysis

We performed a meta-analysis on pooled studies, by test methods (CLIA, LFIA or ELISA) and test antigen (nucleocapsid [N], receptor-binding domain [RBD], N and spike glycoprotein [S], S, subunit 1 of the spike glycoprotein [S1]), which reported sensitivities and specificities for 7 days and overall. The study aims to identify the best test method and or test antigen type, with the highest pooled sensitivity and specificity results, to diagnose SARS-CoV-2 at 7 day and overall post infection. Statistical analysis was performed using R, version 4.0.2 (R Foundation for Statistical Computing, Vienna, Austria) with ‘mada’ package [17]. We performed a bivariate meta-analysis by pooling sensitivities and specificities of test method, and for test method and test antigen. For the 7 days meta-analysis, we analyzed pooled sensitivities, while we used both pooled sensitivities and specificities for the overall analysis. Heterogeneity was assessed based level of statistical significance, where p < 0.05 shows that the true effects vary [18]. Results from the meta-analysis were summarized into pooled mean and 95% CI.

## Results

### Characteristics of the studies

**Figure 1.**
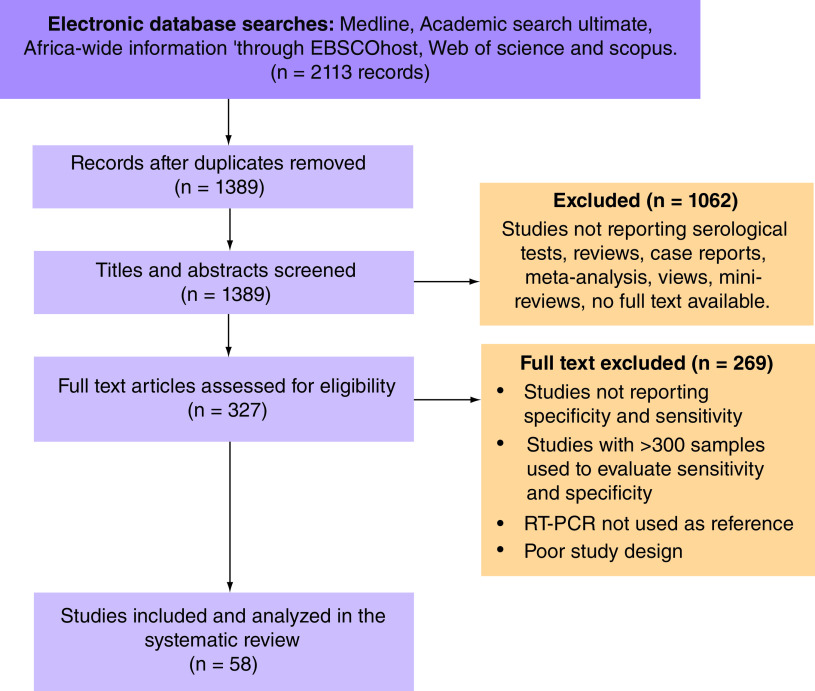
Studies considered and included.

Overall, 2113 records were identified through database searches, and 1389 records were analyzed after duplicate removal. A total of 1062 records were excluded after a full screening of the title and abstract. Finally, 327 full texts were screened, and 58 articles met the inclusion criteria (Figure 1) [19–76]. Table 1 summarizes the studies, including countries, test methods and number of samples. The total number of tests extracted exceeds the total number of publications included in the review because more than one method was evaluated in some studies. The maximum number of tests evaluated in a single study was 12 [29].

**Table 1.** Summary of publications included in the review.

Study	Year	Country	Methods	In-house/ commercial assay	Antigen	Sample size	Ref.
Evaluation of antibody response in symptomatic and asymptomatic COVID-19 patients and diagnostic assessment of new IgM/IgG ELISA kits	2021	Qatar	ELISA	Commercial	S, S1, N	291 serum samples from COVID-19 patients, 119 pre-pandemic serum samples	[19]
A peptide-based magnetic chemiluminescence enzyme immunoassay for serological diagnosis of COVID-19	2020	China	CLIA	Commercial	^†^Synthetic peptide (ORF1a/b, S, N)	200 control samples 177 serum from other infections 276 serum samples	[21]
Detection of SARS-CoV-2-specific antibodies via rapid diagnostic immunoassays in COVID-19 patients	2021	Thailand	LFIA, ELISA, CMIA	In-house	S1, N	245 PCR-positive samples, 130 pre-pandemic samples.	[22]
Comparative evaluation of SARS-CoV-2 IgG assays in India.	2020	India	ELISA, CLIA	One in-house and two commercial assays	S1, S2, RBD	379 COVID-19 samples 184 negative control samples	[23]
Multicenter evaluation of two chemiluminescence and three lateral flow immunoassays for the diagnosis of COVID-19 and assessment of antibody dynamic responses to SARS-CoV-2 in Taiwan.	2020	Taiwan	CLIA, LFIA	Commercial	N, S	346 serum from 74 positive patients; 194 from non-COVID-19 patients	[24]
Clinical evaluation of serological IgG antibody response on the Abbott Architect for established SARS-CoV-2 infection.	2020	Singapore	CLIA	Commercial	N	177 symptomatic COVID-19-positive patients, 163 non-COVID pre-pandemic serum samples.	[25]
Recent advances in the evaluation of serological assays for the diagnosis of SARS-CoV-2 infection and COVID-19	2021	Italy	CLIA, ELISA, LFIA	Commercial	S, S1, S2, N	207 PCR-positive samples and 130 RT-PCR negative	[26]
Validation of a combined ELISA to detect IgG, IgA and IgM antibody responses to SARS-CoV-2 in mild or moderate non-hospitalized patients	2021	UK	ELISA	In-house	S	73 PCR-confirmed COVID-19 patients, 359 sera from COVID-19 patients	[27]
Comparison of SARS-CoV-2 serological tests with different antigen targets	2020	Switzerland	LFA, ELISA, CLIA	Commercial	S, S1, S2, N, whole virus lysate	178 positive PCR samples, 404 negatives (pre-pandemic).	[28]
Diagnostic performance of commercially available COVID-19 serology tests in Brazil.	2020	Brazil	LFIA, ELISA	Commercial	Not specified	289 samples from 173 positive patients, 116 negative controls.	[29]
Comparison of five serological assays for the detection of SARS-CoV-2 antibodies	2021	Germany	CLIA, ELISA	Commercial	S, S1, N	148 PCR positive samples,152 pre-pandemic donors	[30]
Rapid determination of SARS-CoV-2 antibodies using a bedside, point-of-care, serological test.	2020	France	LFIA	Commercial	N	256 COVID-19 samples 50 negative control samples	[31]
Evaluating 10 commercially available SARS-CoV-2 rapid serological tests by use of the STARD method	2021	France	^‡^LFIA	Commercial	S, N^†^	250 serum with documented RT-PCR-positive results and, 254 pre-pandemic serum samples	[32]
A high-throughput anti-SARS-CoV-2 IgG testing platform for COVID-19	2021	USA	Luminex assay	In-house	RBD	107 positive PCR samples, 226 COVID-19-negative samples	[33]
Automated Western immunoblotting detection of anti-SARS-CoV-2 serum antibodies	2021	France	Immunoblot	In-house	S, N	223 sera from COVID-19 patients, 379 non-COVID-19 samples	[34]
Comparison of the Elecsys^®^ Anti-SARS-CoV-2 immunoassay with the EDI™ enzyme linked immunosorbent assays for the detection of SARS-CoV-2 antibodies in human plasma.	2020	Austria	CLIA, ELISA	Commercial	N	104 samples from 64 COVID-19-positive patients; 200 healthy blood donors and 256 samples from ICU patients prior to the COVID outbreak.	[35]
Improved detection of antibodies against SARS-CoV-2 by microsphere-based antibody assay.	2020	China	ELISA	In-house MBA Commercial ELISA	N	39 COVID-19 samples 294 negative control samples	[36]
A comparison of four serological assays for detecting anti-SARS-CoV-2 antibodies in human serum samples from different populations.	2020	France	ELISA S-Flow assay LIPS assay	Commercial ELISA In-house S-Flow and LIPS assay	S, S1, N	51 COVID-19 samples 209 COVID-19 suspected samples 691 negative control samples	[37]
Comparison of the clinical performances of the Abbott Alinity IgG, Abbott Architect IgM and Roche Elecsys Total SARS-CoV-2 antibody assays	2021	USA	CMIA, ELISA	Commercial	N	103 PCR-positive samples, 580 pre-COVID-19 samples	[38]
Performance of three automated SARS-CoV-2 antibody assays and relevance of orthogonal testing algorithms	2020	Belgium	CLIA	Commercial	S1, S2, N	186 samples positive to COVID-19 PCR, 120 pre-pandemic samples.	[39]
Development, performance evaluation and clinical application of a Rapid SARS-CoV-2 IgM and IgG Test Kit based on automated fluorescence immunoassay	2021	China	LFIA	In-house	RBD	733 PCR-positive samples, 223 non COVID-19 samples used as negative control	[40]
Development of an automated chemiluminescence assay system for quantitative measurement of multiple Anti-SARS-CoV-2 antibodies	2021	Japan	CLIA	In-house	S, N	153 serum samples from COVID-19 patients, 1000 serum samples from healthy donors	[41]
Evaluation of 11 SARS-CoV-2 antibody tests by using samples from patients with defined IgG antibody titers	2021	Sweden	IFA, ELISA, CLIA	In-house IFA Commercial ELISAs and CLIA	S, S1, RBD, N	306 sera from COVID-19 patients, 278 pre-pandemic samples	[42]
Prevalence of SARS-CoV-2 infection in health workers and diagnostic test performance: the experience of a teaching hospital in central Italy.	2020	Italy	CLIA	Commercial	S, N	2057 healthcare workers, 58 RT-PCR positive,	[43]
Performance of an automated chemiluminescence SARS-CoV-2 IG-G assay.	2020	Singapore	CMIA	Commercial	N	262 healthcare workers, 718 stored samples from the staff (2018) as controls, 353 COVID-19 samples from stored samples.	[44]
Serum SARS-CoV-2 nucleocapsid protein: a sensitivity and specificity early diagnostic marker for SARS-COV-2 infection.	2020	China	ELISA, LFIA	Commercial	N	633 negative control samples	[45]
Development and clinical application of a rapid IgM‐IgG combined antibody test for SARS‐CoV‐2 infection diagnosis	2020	China	LFIA	In-house	RBD	397 COVID-19 samples 128 negative control samples	[46]
Systematic evaluation of IgG responses to SARS-CoV-2 spike protein-derived peptides for monitoring COVID-19 patients	2021	China	Microarray	In-house	S1	2434 sera from 858 COVID-19 patients, 63 asymptomatic patients and 610 controls	[47]
Development and clinical application of a rapid SARS-CoV-2 antibody test strip: a multi-center assessment across China	2020	China	LFIA	In-house	RBD, N	170 COVID-19 samples 300 negative controls samples	[48]
Multicenter evaluation of four immunoassays for the performance of early diagnosis of COVID-19 and assessment of antibody responses of patients with pneumonia in Taiwan	2021	Taiwan	CLIA, ELISA	Commercial	S, S1, RBD	200 sera from non-COVID-19 patients, 184 sera from COVID-19 patients	[49]
A preliminary study on serological assay for SARS-CoV-2 in 238 admitted hospital patients.	2020	China	ELISA	Commercial	N	153 laboratory-confirmed cases and 85 tested negative; controls: 70 ordinary patients and 50 healthy blood donors.	[50]
Clinical application of chemiluminescence microparticle immunoassay for SARS-CoV-2 infection diagnosis.	2020	China	CMIA	Commercial	RBD	206 COVID-19-positive patients. 270 healthy patients with no other infections or autoimmune diseases.	[51]
Evaluation of nucleocapsid and spike protein-based ELISA for detecting antibodies against SARS-CoV-2.	2020	China	ELISA	Commercial	S, N	214 COVID-19-positive samples and 100 healthy blood donors.	[52]
A facile assay for rapid detection of COVID-19 antibodies	2020	USA, China	LFIA	In-house	N	217 COVID-19 patients 158 negative control samples	[53]
Longitudinal characterization of the IgM and IgG humoral response in symptomatic COVID-19 patients using the Abbott Architect	2020	USA	CMIA	Commercial	RBD, N	1349 sera from COVID-19 patients, 300 sera from pre-pandemic samples	[54]
Evaluation of Abbott anti-SARS-CoV-2 CMIA IgG and Euroimmun ELISA IgG/IgA assays in a clinical lab	2020	USA	CMIA, ELISA	Commercial	S1, N	97 SARS-CoV-2-positive samples; control: 215 COVID-19-negative samples (78 of these had positive serology test results of other infectious diseases or autoimmunity); 847 pre-COVID-19 samples	[55]
Retrospective clinical evaluation of 4 lateral flow assays for the detection of SARS-CoV-2 IgG.	2020	USA	LFIA	Commercial	Not specified	457, 200, 155 samples.	[56]
Development of a fast SARS-CoV-2 IgG ELISA, based on receptor-binding domain, and its comparative evaluation using temporally segregated samples from RT-PCR positive individuals	2021	India	ELISA	In-house and commercial ELISAs	SARS-CoV-2, S1, RBD	470 pre-pandemic samples, 312 sera from SARS-CoV-2 RT-PCR-positive individuals	[57]
Clinical application of combined detection of SARS-CoV-2-specific antibody and nucleic acid	2020	China	LFIA	Commercial	SARS-CoV-2	652 suspected COVID-19 patients and 206 non-COVID patients	[58]
Validation of SARS-CoV-2 serological essay, Bahrain experience	2020	Bahrain	CLIA	Commercial	N	388 serum samples	[59]
Comparison of serologic and molecular SARS-CoV-2 results in a large cohort in southern Tuscany demonstrates a role for serologic testing to increase diagnostic sensitivity.	2020	Italy	LFIA	Commercial	Not specified	516 samples (413 SARS-CoV-2 negative, 73 positive, 25 undetermined, 5 invalids)	[60]
Diagnostic accuracy comparison of three fully automated chemiluminescent immunoassay platforms for the detection of SARS-CoV-2 antibodies	2021	India	CLIA, CMIA	Commercial	S, N	Serum samples of 594 COVID-19 positive patients and 100 samples from pre-COVID-19 cases	[61]
Comparative performance of five commercially available serologic assays to detect antibodies to SARS-CoV-2 and identify individuals with high neutralizing titers	2021	USA	ELISA, CMIA, eCLIA	Commercial	S1, N	214 PCR-positive samples. 1099 pre-pandemic samples	[62]
Clinical evaluation of five different automated SARS-CoV-2 serology assays in a cohort of hospitalized COVID-19 patients.	2020	Germany	ELISA, CLIA	Commercial	S, S1	75 COVID-19-positive patients, 320 pre-pandemic COVID-19-negative samples.	[63]
Performance of the COVID19SEROSpeed IgM/IgG rapid test, an immunochromatographic assay for the diagnosis of SARS-CoV-2 infection: a multicenter European study	2021	Germany, France and Italy	LFIA	Commercial	S1, S2, RBD, N, inactivated native antigen	564 PCR-positive samples. 215 pre-pandemic serum	[64]
Evaluation of performance of two SARS-CoV-2 rapid IgM-IgG combined antibody tests on capillary whole blood samples from the fingertip.	2020	France	LFIA	Commercial	N	238 COVID-19-postive patients, 143 COVID-19-negative patients	[65]
Development and multicenter performance evaluation of fully automated SARS-CoV-2 IgM and IgG immunoassays	2020	China	CLIA	In-house	S, N	972 control samples; 513 COVID-19-positive confirmed patients	[66]
Multiplex assays for the identification of serological signatures of SARS-CoV-2 infection: an antibody-based diagnostic and machine learning study	2021	France	Luminex assay	In-house	S, N	259 PCR-positive samples, 335 pre-pandemic samples	[67]
Validity of a serological diagnostic kit for SARS-CoV-2 available in Iran.	2020	Iran	LFIA	Commercial	Not specified	114 COVID-19-positive patients, 198 negative sera	[68]
Comparative clinical evaluation of the Roche Elecsys and Abbott SARS-CoV-2 serology assays for COVID-19	2021	Singapore	CLIA, CMIA	Commercial	N	COVID-19 confirmed patients (n = 170) and negative controls (n = 163) obtained before December 2019,	[69]
Performance characteristics of four high-throughput immunoassays for detection of IgG antibodies against SARS-CoV-2.	2020	USA	ELISA, CMIA, CLIA	Commercial	S, S1, N	224 COVID-19 samples 254 control samples	[70]
Evaluation of three commercial SARS-CoV-2 serologic assays and their performance in two-test algorithms.	2020	USA	ELISA, CMIA, eCLIA	Commercial	S1, S2, N	128 symptomatic COVID-19-positive patients; 1204 pre-pandemic samples; 64 COVID-19-negative samples (PCR) with respiratory symptoms	[71]
Antibody response against SARS-CoV-2 spike protein and nucleoprotein evaluated by four automated immunoassays and three ELISAs.	2020	Belgium	CLIA, ELISA	Commercial	S, S1, S2, N	113 patients collected before January 2020 as negative controls, 24 samples from patients with a confirmed non-SARS-CoV-2 infection collected 12–42 days after positive PCR. 233 samples of 114 patients who were positive for SARS-CoV-2 with RT-PCR	[72]
Evaluation of the performance of SARS-CoV-2 serological tools and their positioning in COVID-19 diagnostic strategies.	2020	France	LFIA, ELISA	Commercial	S1, RBD, N	325 samples: panel 1–55 hospitalized patients. Panel 2–143 healthcare workers. 100 pre-pandemic samples. 20 anti-hCov positive samples.	[73]
Combination of serological total antibody and RT-PCR test for detection of SARS-CoV-2 infections.	2020	China	CMIA	Commercial	RBD	375 patients who visited the hospital with respiratory complaints were included. Of the patients, 141 were confirmed to have SARS-CoV-2 infection (COVID-19 group), the other 234 patients with no relevance to COVID-19 were included in a control group	[74]
Characteristics of three different chemiluminescence assays for testing for SARS-CoV-2 antibodies	2021	Switzerland	eCLIA, CMIA, LIA	Commercial	S1, S2, N	145 COVID-19 patients whose serum was drawn after COVID-19 disease was confirmed by RT-PCR and used to determine sensitivity. Specificity was evaluated using 191 healthy blood donors and 1002 healthy workers	[75]
Evaluation of serum IgM and IgG antibodies in COVID‐19 patients by ELISA	2020	China	ELISA	In-house	S	150 serum samples from COVID-19 patients, 150 serum samples from non-COVID-19 patients	[76]

† Only S was used to screen samples.

‡ Target antigens reported for two out of the ten assays evaluated: NG-Test IgG-IgM COVID-19 (NG-Biotech, Guipry, France) (RDT 1); a 2019-nCoV Ab test (Innovita Biological Technology Co., Qian'an, China) (RDT 6).

CLIA: Chemiluminescence immunoassay; LFIA: Lateral flow immunochromatographic assay; LIPS: Luciferase immunoprecipitation system; ORF: Open reading frame; RBD: Receptor-binding domain; STARD: Standards for Reporting of Diagnostic Accuracy Studies.

The global distribution of identified articles include China (n = 14), the USA (n = 10), France (n = 8), Italy (n = 3), Singapore (n = 3), Germany (n = 3), India (n = 3), Taiwan (n = 2), Belgium (n = 2), while Switzerland, Austria, Bahrain, Brazil, Iran, Italy, Japan, Sweden, Thailand, Qatar and the United Kingdom each contributed one article. Two of the selected studies were conducted in more than one country [53,64].

A range of serological assays were evaluated in the identified articles and these include CLIA (n = 64), ELISA (n = 49), LFIA (n = 31), luciferase immunoprecipitation system (n = 3), Immunoblot and flow cytometry-based assays were each reported in single studies. One study used synthetic peptides derived from the open reading frame 1a/b, S and N proteins to develop a chemiluminescent assay to detect IgM and IgG against SARS-CoV-2 [21]. The antigen types described in the articles were N (n = 77), S (n = 28), S1 (n = 25), RBD (n = 15), subunit 2 of the spike glycoprotein (S2 [ n = 8]) and whole virus lysate (n = 4). In four articles, the target antigen was not reported.

Forty-four papers reported diagnostic data on commercial serological kits while 18 studies reported data obtained using in-house developed kits (Table 1). In five publications, in-house assays were evaluated in parallel with commercial assays [23,36,37,42,57].

### Sensitivity from onset of symptoms to 7 days

We identified 22 articles that reported the sensitivity data from onset of symptoms up to 7 days post symptom onset and extracted data on sensitivity, the antigens targeted, and the antibodies detected (Supplementary Table 6). Specificity data based on days after onset of symptoms was not reported in the identified articles. In three articles [59,72,73], sensitivity and specificity data were stratified by days after PCR test and were therefore not included in the analysis.

A total of 60 serology methods were extracted including CLIA (n = 26), LFIA (n = 17), ELISA (n = 16) and flow cytometry (n = 1). A total of 61 results were extracted and analyzed since some of the methods gave more than one result for immunoglobulin type detection (Supplementary Table 6). Eight antigen formats were described in the assays and the most frequently evaluated immunoglobulin classes included IgG (n = 36) and IgM (n = 16). Three studies evaluated IgA and reported sensitivities of 37.5 [22], 33.3 [29] and 23% [73]. A Luminex-based assay using the RBD antigen reported a positive percent agreement of 46.15% [33]. We also identified one study [49] reporting a total antibody-based fluorescence enzyme immunoassay system using S1 antigens with 53.9% sensitivity.

**Figure 2.**
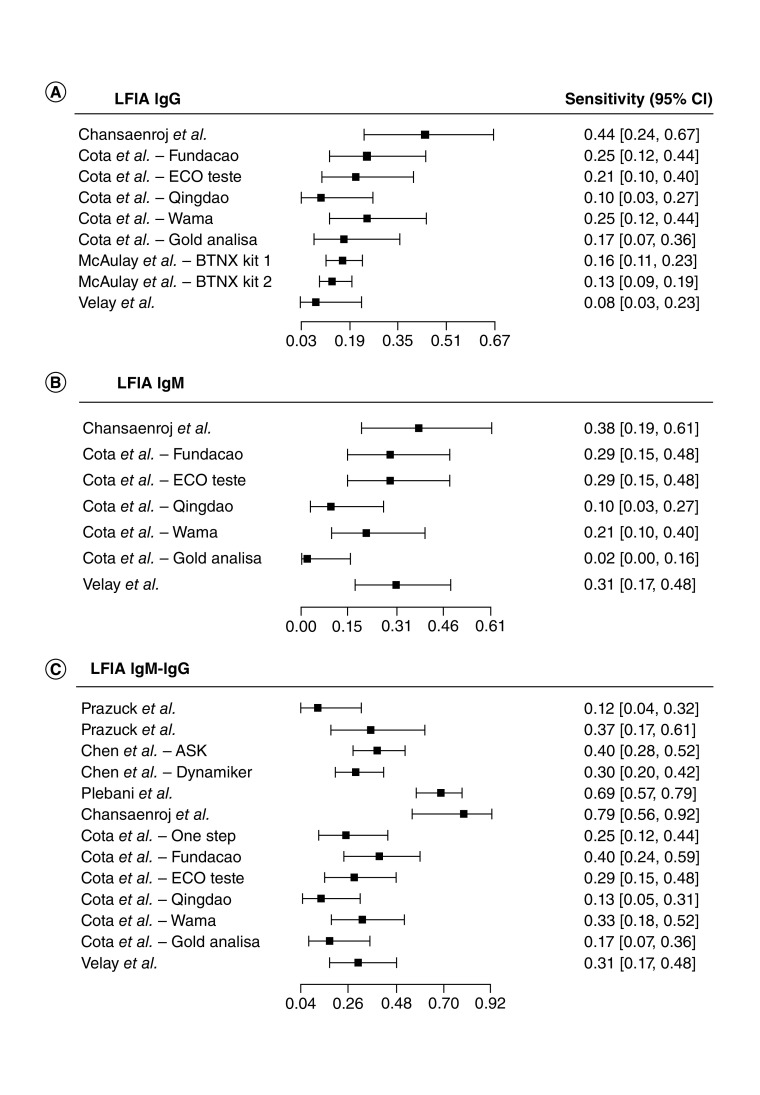
Forest plot of sensitivity for lateral flow immunochromatographic assay serological diagnosis of COVID-19. **(A)** LFIA IgG tests. **(B)** LFIA IgM tests. **(C)** LFIA IgM-IgG tests. LFIA: Lateral flow immunochromatographic assay.

The forest plots in Figure 2 show the sensitivity range for the LFIA serological tests detecting SARS-CoV-2 antibodies in the identified articles. The sensitivity of IgG based LFIA tests (n = 9), ranged from 0.08 (95% CI: 0.03–0.23) [73] to 0.44 (95% CI: 0.24–0.67) [29]. The LFIA IgM tests (n = 7) sensitivity ranged from 0.02 (95% CI: 0–0.16) to 0.38 (95% CI: 0.19–0.61) [22]. While the LFIA IgM-IgG tests (n = 13), sensitivity ranged from 0.12 (95% CI: 0.04–0.32) [65] to 0.79 (95% CI: 0.56–0.92) [22].

**Figure 3.**
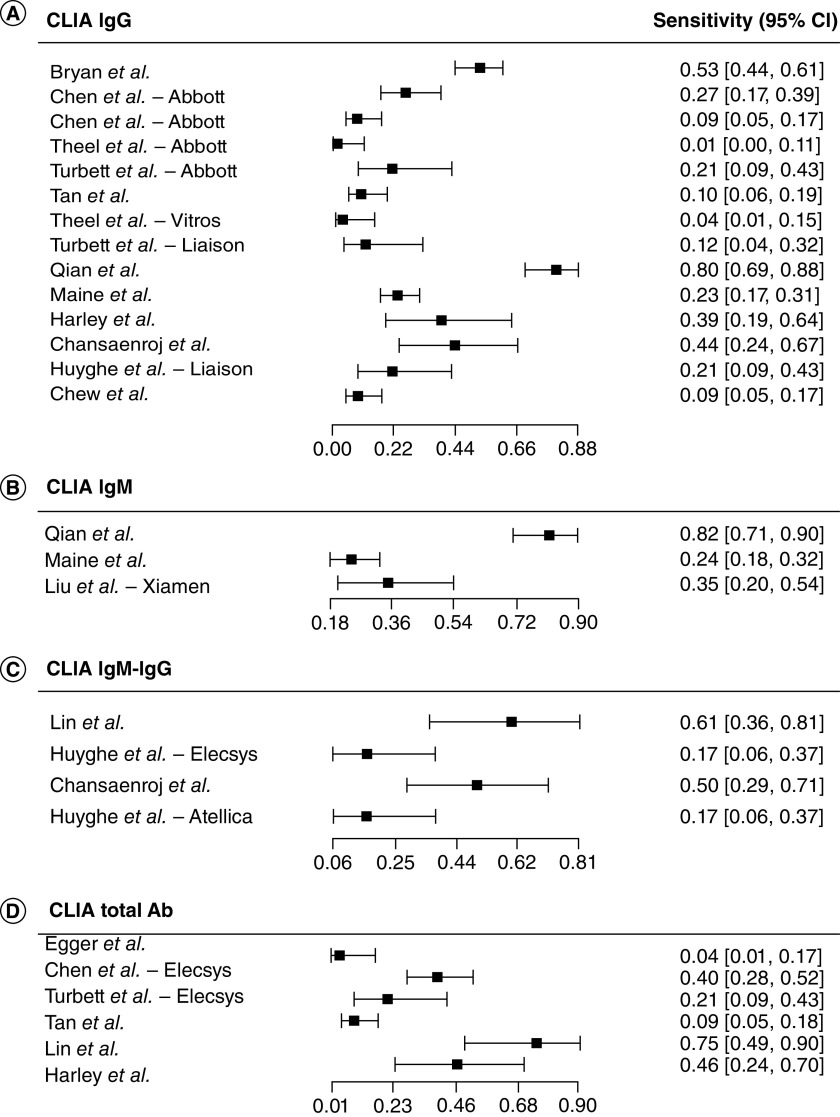
Forest plot of sensitivity for chemiluminescence immunoassay serological diagnosis of COVID-19. **(A)** CLIA IgG tests. **(B)** CLIA IgM tests. **(C)** CLIA IgM-IgG tests. **(D)** CLIA Total antibody tests. CLIA: Chemiluminescence immunoassay.

**Figure 4.**
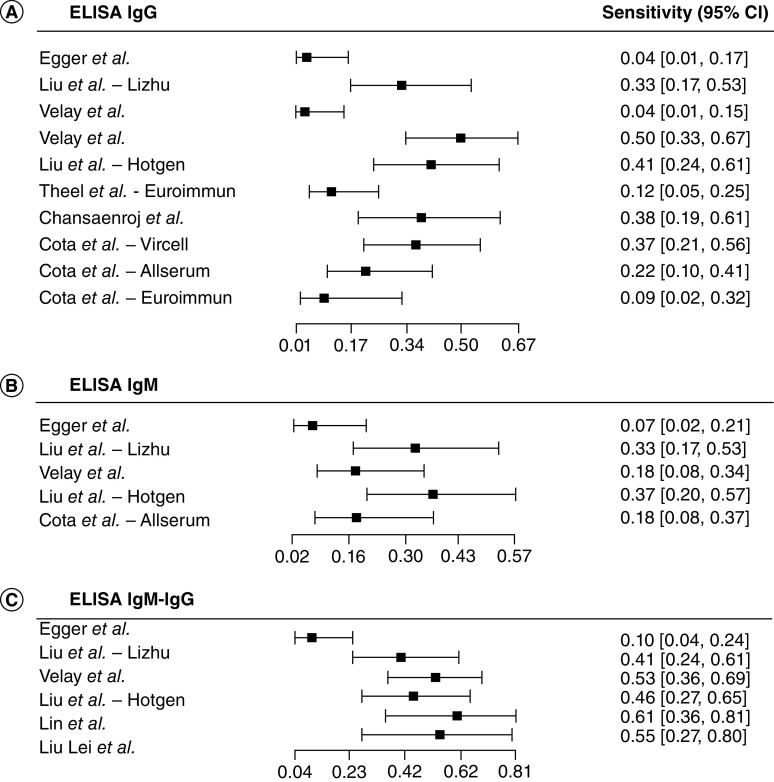
Forest plot of sensitivity for ELISA diagnosis of COVID-19. **(A)** ELISA IgG tests. **(B)** ELISA IgM tests. **(C)** ELISA IgM-IgG tests.

We then evaluated the performance of the CLIA compared with the rRT-PCR (Figure 3). The sensitivity estimates of CLIA IgG tests (n = 14) ranged from 0.01 (95% CI: 0.00–0.11) [70] to 0.80 (95% CI: 0.69–0.88) [66]. The sensitivity for the CLIA IgM or IgM-IgG tests (n = 7) ranged from 0.17 (95% CI: 0.06–0.37) [39] to 0.82 (95% CI: 0.71–0.90) [66]. The CLIA total antibody-based tests (n = 6) sensitivity estimates ranged from 0.04 (95% CI: 0.01–0.17) [35] to 0.75 (95% CI: 0.49–0.90) [49]. At last, we analyzed the performance of the ELISA compared with the rRT-PCR (Figure 4). Among the ELISA IgG tests (n = 10), sensitivity ranged from 0.04 (95% CI: 0.01–0.15) to 0.50 (95% CI: 0.33–0.67) [73]. ELISA IgM tests (n = 5), sensitivity ranged from 0.07 (95% CI: 0.02–0.21) [35] to 0.37 (95% CI: 0.20–0.57) [52], while the ELISA IgM-IgG based tests (n = 6), sensitivity ranged from 0.10 (95% CI: 0.04–0.24) [35] to 0.61 (95% CI: 0.36–0.81) [49].

We performed a meta-analysis to evaluate the performance of the serological tests within 7 days of post symptoms onset and the results are shown in Table 2. The pooled sensitivity for the IgG, IgM and IgM-IgG based LFIA tests were 20% (95% CI: 10.15–35.82), 22.8% (95% CI: 11.42–41.19) and 35% (95% CI: 21.65–52.04), respectively. The pooled sensitivity for the IgG, IgM and IgM-IgG based CLIA tests were 25.4% (95% CI: 16.29–39.09), 47.2% (95% CI: 36.3–58.64) and 36% (95% CI: 19.18–56.84), respectively. The pooled sensitivity for the IgG, IgM and IgM-IgG based ELISA tests were 25% (95% CI: 13.39–42.83), 22.5% (95% CI: 11.13–40.42) and 44.3% (95% CI: 25.72–63.5), respectively. We also evaluated whether targeting a particular antigen or combining antigens results in higher serological test performance and the results are shown in Table 3. The obtained sensitivity ranged from 19.2% (95% CI: 9.18–36) to 53.2% (95% CI: 31.52–73.16). However, there was no significant difference among the pooled mean sensitivities obtained by the serological tests (p > 0.05).

**Table 2.** Estimates of test accuracy within 7 days of symptom onset.

Test method and antibody type	Studies (n)	Sensitivity (%) (95% CI)
CLIA IgG	14	25.4 (16.29–39.09)
CLIA IgM	3	47.2 (36.3–58.64)
CLIA IgM-IgG	4	36 (19.18–56.84)
LFIA IgG	9	20 (10.15–35.82)
LFIA IgM	7	22.8 (11.42–41.19)
LFIA IgM-IgG	13	35 (21.65–52.04)
ELISA IgG	10	25 (13.39–42.83)
ELISA IgM	5	22.5 (11.13–40.42)
ELISA IgM-IgG	6	44.3 (25.72–63.5)

CLIA: Chemiluminescence immunoassay; LFIA: Lateral flow immunochromatographic assay.

**Table 3.** Sensitivity at 7 days as per the test antigen.

Classification	Studies (n)	Sensitivity (%) (95% CI)
CLIA IgG N	9	23.8 (14.22–37.6)
CLIA IgM-IgG N	2	33.3 (17.41–54.41)
CLIA Total antibody N	5	24.2 (13.43–39.81)
ELISA IgG N	4	22.7 (13.12–37.83)
ELISA IgG S1	2	24.9 (12.12–43.27)
ELISA IgM N	3	19.2 (9.18–36)
ELISA IgM-IgG N	4	39.9 (22.82–58.68)
ELISA IgM-IgG S	2	53.2 (31.52–73.16)
LFIA IgM-IgG N	5	45.3 (30.82–61.2)
LFIA IgM-IgG S	2	35.1 (22.81–50.09)

CLIA: Chemiluminescence immunoassay; LFIA: Lateral flow immunochromatographic assay; N: Nucleocapsid; S: Spike glycoprotein; S1: Subunit 1 of the spike glycoprotein.

We next evaluated heterogeneity for the LFIA, CLIA and ELISA based methods (Supplementary Table 1). Significant heterogeneity was observed for all tests except the CLIA IgM-IgG, ELISA IgG, ELISA IgM-IgG and LFIA IgM-IgG tests.

### Overall sensitivity

We identified 33 articles that reported the overall sensitivity and specificity (Supplementary Table 7). A total of 73 serology methods were extracted consisting of CLIA (n = 33), ELISA (n = 27), LFIA (n = 10) and one each for flow cytometry, microarray and immunoblot based assays. Sensitivities reported with the luciferase immunoprecipitation assay [37], peptide assay based on the S antigen [47] and the immunoblot assay [34] were 69, 95.5 and 81%, respectively, while the specificities ranged from 93 to 99%. Results also suggest that combining S and N as the target antigen performs better than any other antigen or combination of antigens.

**Figure 5.**
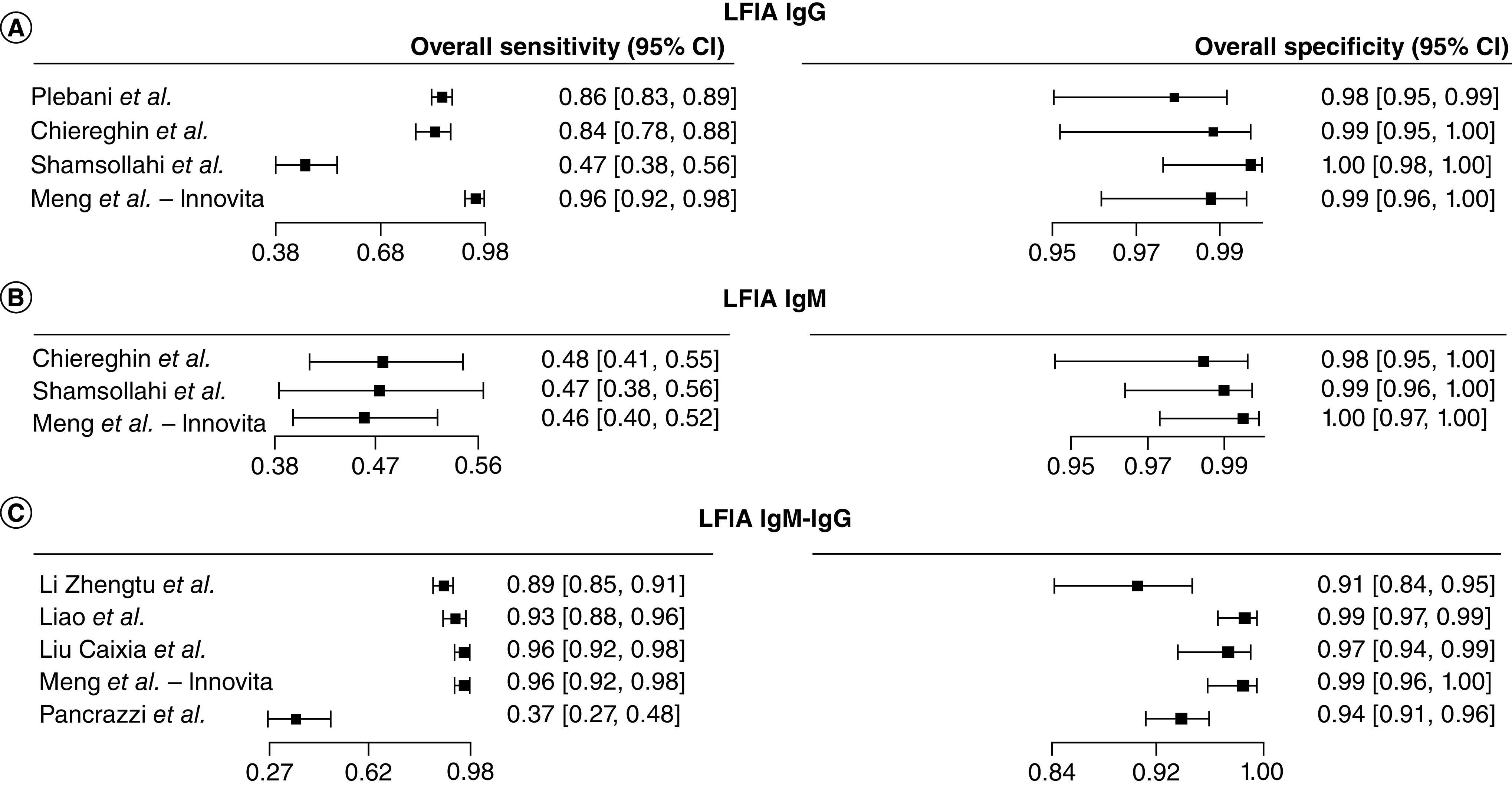
Forest plot of sensitivity and specificity for lateral flow immunochromatographic assay serological diagnosis of COVID-19. **(A)** LFIA IgG tests. **(B)** LFIA IgM tests. **(C)** LFIA IgM-IgG tests. LFIA: Lateral flow immunochromatographic assay.

**Figure 6.**
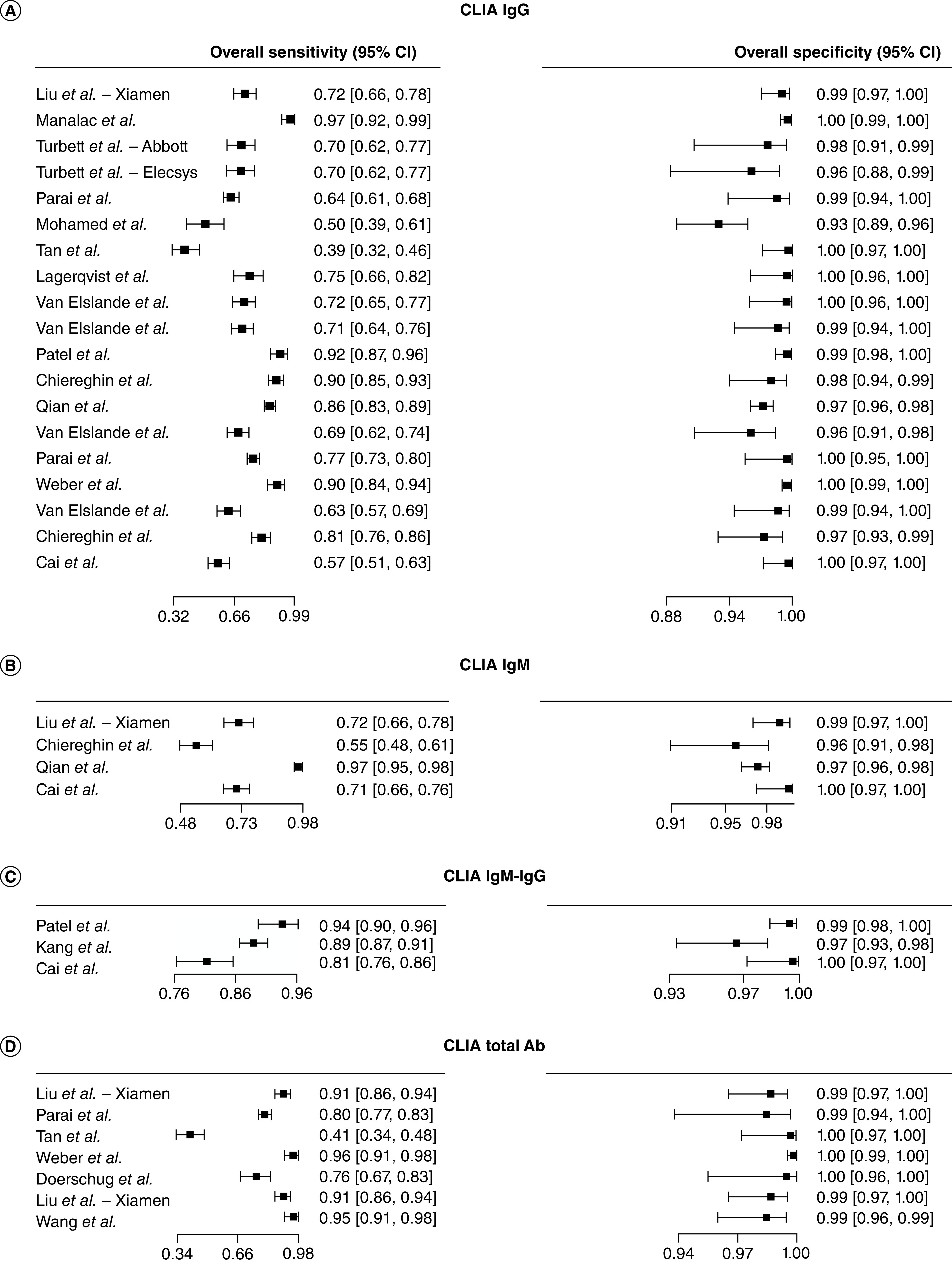
Forest plot of sensitivity and specificity for chemiluminescence immunoassay serological diagnosis of COVID-19. **(A)** CLIA IgG tests. **(B)** CLIA IgM tests. **(C)** CLIA IgM-IgG tests. **(D)** CLIA Total antibody tests. CLIA: Chemiluminescence immunoassay.

**Figure 7.**
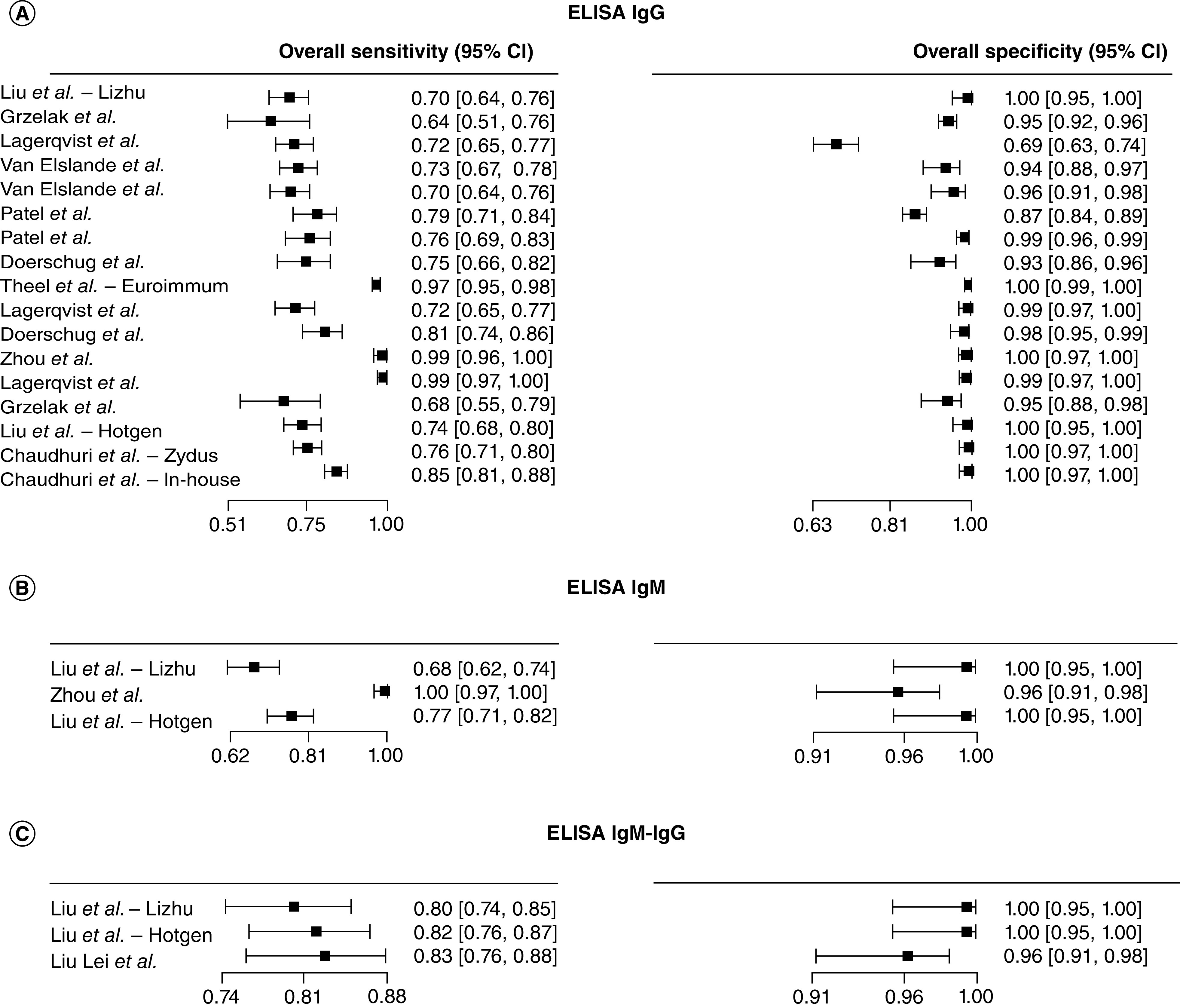
Forest plot of sensitivity and specificity for ELISA diagnosis of COVID-19. **(A)** ELISA IgG tests. **(B)** ELISA IgM tests. **(C)** ELISA IgM-IgG tests.

The overall sensitivity for the IgG, IgM and IgM-IgG LFIA tests (n = 12) ranged from 0.37 (95% CI: 0.27–0.48) [60] to 0.96 (95% CI: 0.92–0.98) [53,58] and specificity from 0.91 (95% CI: 0.84–0.95) [46] to 1 (95% CI: 0.98–1) (Figure 5) [68]. Among the CLIA tests (n = 33), the sensitivity estimates spanned from 0.39 (95% CI: 0.32–0.46) [69] to 0.97 (95% CI: 0.92–0.99) [55] and specificity from 0.93 (95% CI: 0.89–0.96) [71] to 1.00 (95% CI: 0.95–1.00) (Figure 6) [61]. The overall sensitivity estimates for the ELISA tests (n = 23) ranged from 0.64 (95% CI: 0.51–0.76) [37] to 1.00 (95% CI: 0.97–1.00) [76] and specificity from 0.69 (95% CI: 0.63–0.74) [42] to 1.00 (95% CI: 0.99–1.00) (Figure 7) [70].

The overall performance of serological tests compared with the rRT-PCR was evaluated and the results are shown in Table 4. The pooled sensitivity for the IgG, IgM and IgM-IgG based LFIA tests were 78% (95% CI: 72.71–2.48), 47.1% (95 CI: 39.77–54.48) and 82% (95% CI: 76.93–86.27), respectively. The pooled sensitivity for the IgG, IgM and IgM-IgG based CLIA tests were 72.9% (95% CI: 66.64–78.22), 73.7% (95% CI: 68.47–78.31) and 88.1% (95% CI: 84.26–91.01), respectively. The pooled sensitivity for the IgG, IgM and IgM-IgG based ELISA tests were 78.2% (95% CI: 71.68–83.53), 81.6% (95% CI: 76.48–85.36) and 81.7% (95% CI: 75.62–86.53), respectively. The specificities for all the tests were high ranging from 95.3 to 99%. Additionally, we evaluated the overall performance of the serological tests as per the targeted antigens (Table 5). The obtained sensitivity ranged from 70.2% (95% CI: 63.24–76.18) to 93.3% (95% CI: 88.54–96.03) while specificity spanned from 87.5% (95% CI: 80.83–92.09) to 99.6% (95% CI: 96.45–99.96) (Table 5).

**Table 4.** Overall estimates of test accuracy.

Test method and antibody type	Studies (n)	Sensitivity (%) (95% CI)	Specificity (%) (95% CI)
CLIA IgG	19	72.9 (66.64–78.22)	98.3 (94.72–99.32)
CLIA IgM	4	73.7 (68.47–78.31)	98 (95.33–99)
CLIA IgM-IgG	3	88.1 (84.26–91.01)	98.6 (96.34–99.38)
LFIA IgG	4	78 (72.71–82.48)	98.8 (96.01–99.62)
LFIA IgM	3	47.1 (39.77–54.48)	99 (96.09–99.74)
LFIA IgM-IgG	5	82 (76.93–86.27)	95.9 (92.33–97.68)
ELISA IgG	17	78.2 (71.68–83.53)	95.3 (91.75–96.9)
ELISA IgM	3	81.6 (76.48–85.36)	98.2 (94.01–99.28)
ELISA IgM-IgG	3	81.7 (75.62–86.53)	98.4 (94.04–99.46)

CLIA: Chemiluminescence immunoassay; LFIA: Lateral flow immunochromatographic assay.

**Table 5.** Overall sensitivity and specificity as per the test antigen.

Classification	Studies (n)	Overall sensitivity (%) (95% CI)	Overall specificity (%) (95% CI)
CLIA IgG N	11	70.2 (63.24–76.18)	98.3 (94.52–99.33)
CLIA IgG N&S	4	80.2 (75.73–84.02)	97.7 (94.06–98.96)
CLIA IgG S1&S2	3	78.4 (72.25–83.22)	98.8 (95.91–99.64)
CLIA IgM N&S	2	75.6 (71.27–79.57)	96.8 (93.77–98.3)
CLIA Total antibody N	5	76.6 (70.86–81.27)	99.3 (96.49–99.81)
CLIA Total antibody RBD	2	93.3 (88.54–96.03)	98.8 (96.54–99.59)
ELISA IgA S1	2	79.3 (72.56–84.77)	87.5 (80.83–92.09)
ELISA IgG N	8	72.4 (64.57–79.06)	91.4 (87.06–93.88)
ELISA IgG RBD	2	79.4 (74.29–83.73)	99.6 (96.45–99.96)
ELISA IgG S	3	88.8 (82.57–92.93)	97.8 (94.11–99.08)
ELISA IgG S1	3	83.3 (78.34–87.31)	99.2 (97.14–99.78)
ELISA IgM-IgG N	2	81.5 (75.23–86.47)	97.9 (93.34–99.21)
LFIA IgG N	2	85 (80.62–88.49)	98.7 (95.54–99.57)

CLIA: Chemiluminescence immunoassay; LFIA: Lateral flow immunochromatographic assay; N: Nucleocapsid; S: Spike glycoprotein; S1: Subunit 1 of the spike glycoprotein; S2: Subunit 2 of the spike glycoprotein; RBD: Receptor-binding domain.

We also evaluated heterogeneity for the LFIA, CLIA and ELISA based methods (Supplementary Table 2). Significant heterogeneity was observed only with the CLIA IgG, ELISA IgG, ELISA IgM-IgG tests.

## Discussion

COVID-19 continues to pose a major global healthcare challenge despite the accelerated delivery of vaccination. It remains necessary to identify infection early and isolate the infected individual to reduce the spread of the disease.

In this systematic review and meta-analysis, we investigated the utility of serological assays for the diagnosis of COVID-19 during the first week of symptom development in PCR-positive patients. Studies reporting sensitivity and specificity data based on days after PCR test to categorize days of symptom onset were not incorporated into the analysis. We reasoned that the actual number of days post symptom onset are likely to be underestimated if classified by the number of days after the PCR test. Additionally, we evaluated the overall diagnostic performance of serological test methods for detecting SARS-CoV-2 antibodies in serum from patients with a positive RT-PCR test.

Our meta-analysis yielded high specificities ranging from 95.3% (95% CI: 91.75–96.9) to 99% (95% CI: 96.09–99.71) compared with the rRT-PCR. Similar studies have reported pooled specificities spanning from 95% (95% CI: 91–98) to 99.9% (97.78–100) [14,15,77,78]. Sensitivity estimates for within 7 days since onset of disease could not be evaluated because identified studies did not provide the data.

Overall, CLIA IgM-IgG demonstrated superior diagnostic accuracy with sensitivity and specificity of 88.1% (95% CI: 84.26–91.01) and 98.6% (95% CI: 96.34–99.38) respectively. Since the 95% CIs were overlapping, we performed statistical analysis of the pooled mean sensitivities to determine whether they were significantly different across the different sets of tests used. The pooled mean sensitivity obtained with CLIA IgM-IgG was significantly higher than LFIA IgM and CLIA IgG only (Supplementary Table 4). Unlike previous studies that have reported lower sensitivities with the LFIA test method compared with the CLIA and ELISA based assays within each antibody class [14,15,77,78], our results show that LFIA tests do have a role to play in detecting antibodies to SARS-CoV-2. While LFIA IgM, 47.1% (95% CI: 39.77–54.48), had the lowest sensitivity: LFIA IgG, 78 % (95% CI: 72.71–82.48), performed marginally better than either CLIA IgG or IgM assays. LFIA IgM-IgG sensitivity of 82% (95% CI: 76.93–86.27) was second only to CLIA IgM-IgG, 88.1% (95% CI: 84.26–91.01). Our results thus show that LFIA IgG and IgM-IgG sensitivities are comparable to the ELISA and CLIA test methods. These LFIA tests could prove useful in resource-limited settings without access to testing laboratories.

Within 7 days of onset of symptoms, the highest pooled sensitivity was obtained with CLIA IgM, 47.2% (95% CI: 36.3–58.64) and ELISA IgM-IgG, 44.3% (95% CI: 25.72–63.5) compared with rRT-PCR. Previous meta-analysis reported pooled sensitivities ranging from 0 to 53.2% (95% CI: 28.7–67.6) [14,77,78]. Our results and those from previous studies demonstrate that the clinical utility of serological assays for the detection of SARS-CoV-2 antibodies within 7 days since symptom onset is limited. Patients with 7 days and fewer post symptoms onset could be in a pre-seroconversion state; consequently, higher antibody positivity has been reported at least 14 days post symptoms onset [78–80].

We evaluated the immunoglobulin class and antigens that can be targeted to maximize the performance of serological tests. Overall, CLIA based assays measuring the total antibody against the RBD had the best diagnostic accuracy with sensitivity and specificity of 93.3% (95% CI: 88.54–96.03) and 98.8% (95% CI: 96.54–99.59), respectively (Table 5). A previous study reported the highest sensitivity with tests detecting the total antibody against both the N and S antigens [78]. Our search did not yield serological tests detecting the total antibody against the N and S antigens. Within 7 days of developing symptoms, the highest sensitivity was obtained with ELISA IgM-IgG targeting the spike protein, 53.2% (95% CI: 31.52-73.16) (Table 3). Even though the CLIA total antibody RBD and the ELISA IgM-IgG S had higher estimates of accuracy, the estimated pooled mean sensitivities and specificities were not significantly higher than all the identified test methods targeting other antigens (Supplementary Table 5).

We assessed whether serological tests detecting both IgM and IgG have higher diagnostic sensitivity compared with tests measuring either IgM or IgG. Such knowledge would assist in prioritizing the procurement of serological diagnostic kits. Our study shows that combining IgG and IgM yields higher sensitivity compared with measuring IgM alone with ELISA tests and IgG in LFIA tests within 7 days of symptom onset (Supplementary Table 3). There was no enhanced benefit of assaying both IgM and IgG with the CLIA based assays during the first week of symptom onset. Overall, detecting both IgM and IgG was superior to detecting IgG or IgM with the CLIA and LFIA tests (Supplementary Table 4). However, larger studies would be required to verify these results.

The performance of IgM-based assays was evaluated against the IgG based serological tests in our study. Regarding overall estimates of diagnostic accuracy, CLIA IgM and ELISA IgM serological methods had higher pooled sensitivity estimates compared with the CLIA IgG and ELISA IgG test methods. In LFIA tests, detecting the IgG seemed to be a better choice than assaying the IgM. The meta-analysis by Bastos *et al.* and Vengesai *et al.* reported higher pooled sensitivities with the CLIA IgG and LFIA IgG based serological assays compared with the respective IgM test methods albeit with overlapping 95% CIs, while with the ELISA methods higher positivity was obtained by detecting the IgM [14,15]. Regarding sensitivity within 7 days of symptom onset, our findings show that neither IgM nor IgG was a more sensitive marker for COVID-19 diagnosis. That IgM and IgG sensitivities were comparable within the first week of disease onset was expected since both have been detected during the first week of symptom onset [8,81,82]. The early appearance of the IgG may be a consequence of the original antigenic sin effect [83]. IgA has been reported early after infection [84,85] thus is a potential early diagnostic marker for SARS-CoV-2. However, few studies have systematically evaluated IgA in large studies. In our study, we identified only three studies reporting sensitivities of 37.5 [22], 33.3 [29] and 23% [73] within the first week of illness.

This study has some limitations. The magnitude of immune response is influenced by several factors such as age, disease severity and the presence of immunodeficiency disorders which were not considered among the study participants from which blood samples were collected. Studies stratifying the study population according to age, disease severity and immune health are therefore necessary. Studies included in the analysis stratified patients according to the date of symptom onset, relying on participants recalling from memory thus potentially introducing recall bias. In addition, there was insufficient data in the studies to evaluate the cross-reactivity of the serological assays.

## Conclusion

Choice of antigen did not appear to influence the outcome of sensitivity although there were differences in sensitivity for different types of assays performed. Our results show that serological tests based on CLIA IgM and ELISA IgM-IgG were the most sensitive during the first week post symptoms onset. As mentioned previously, the role of antibody detection has limitations in acute diagnosis because of the time taken for the development of an endogenous demonstrable antibody response. However, in resource-challenged settings, serology could play a role taking into consideration the limitations of sensitivity when interpreting the results.

**Summary points**

- The present systematic review presents a synthesis of the sensitivity of antibody tests commonly used to diagnose COVID-19. It also assesses which antibody tests could be used to support RT-PCR for the diagnostic of COVID-19.
- Research articles describing or comparing antibody tests for the diagnostic of COVID-19, were extracted from various databases. Those including at least 300 samples tested were included in this study. The sensitivity data were extracted at different time points post positive RT-PCR diagnostic, and a meta-analysis was performed.
- The data showed that measuring IgG and IgM yields higher sensitivity compared with measuring IgM alone with ELISA and IgG in lateral flow immunochromatographic assay tests within 7 days of symptom onset and the highest pooled sensitivities were obtained with IgM-IgG and chemiluminescence immunoassay IgM tests within the first 7 days.
- Serological tests have low sensitivity within the first week of symptom onset and cannot replace nucleic acid amplification tests. However, serological assays can be used to support nucleic acid amplification tests and have application in surveillance. Serological tests based on chemiluminescence immunoassay IgM and ELISA IgM-IgG were the most sensitive during the first week post symptom onset.
