# Supplementary material for: A systematic review and meta-analysis of the sensitivity of antibody tests for the laboratory confirmation of COVID-19
Source: Future Virol. 2021 Dec 15:10.2217/fvl-2021-0211. doi: 10.2217/fvl-2021-0211 (PMC8686841; doi:10.2217/fvl-2021-0211)
Supplement: Supplementary file 1 [file supplementary_tables_1-7.docx]

Table S1: Antibody type within 7 days of onset of symptoms subgroup meta-analysis heterogeneity

| **Test method and antibody type** | **Number of studies** | **P value** |
| --- | --- | --- |
| CLIA IgG N | 9 | 0 |
| CLIA IgM-IgG N | 2 | 0.0647 |
| CLIA Total antibody N | 5 | 0 |
| ELISA IgG N | 4 | 0 |
| ELISA IgG S1 | 2 | 0.0516 |
| ELISA IgM N | 3 | 0.0437 |
| ELISA IgM-IgG N | 4 | 0.001 |
| ELISA IgM-IgG S | 2 | 0.5824 |
| LFIA IgM-IgG N | 5 | 0 |
| LFIA IgM-IgG S | 2 | 0.5425 |

N, nucleocapsid; S, spike glycoprotein; S1, subunit 1 of the spike glycoprotein; S2, subunit 2 of the spike glycoprotein.

Table S2: Overall antibody type subgroup meta-analysis heterogeneity

| **Test method and antibody type** | **Number of studies** | **Sensitivity (p value)** | **Specificity (p value)** |
| --- | --- | --- | --- |
| CLIA IgG N | 11 | 0 | 0 |
| CLIA IgG N&S | 4 | 0 | 0.4054 |
| CLIA IgG S1&S2 | 3 | 0 | 0.0145 |
| CLIA IgM N&S | 2 | 0 | 0.5813 |
| CLIA Total antibody N | 5 | 0 | 0.0333 |
| CLIA Total antibody RBD | 2 | 0.122 | 1 |
| ELISA IgA S1 | 2 | 0.0336 | 0.0061 |
| ELISA IgG N | 8 | 0.423 | 0 |
| ELISA IgG RBD | 2 | 0.0028 | 1 |
| ELISA IgG S | 3 | 0 | 0.0026 |

N, nucleocapsid; S, spike glycoprotein; S1, subunit 1 of the spike glycoprotein; S2, subunit 2 of the spike glycoprotein; RBD, receptor-binding domain.

Table S3: Comparison of sensitivity at 7 days*

| **Classification 1** | **Classification 2** | **Sensitivity (p value)** |
| --- | --- | --- |
| ELISA IgM | ELISA IgM-IgG | 0.0426 |
| ELISA IgM-IgG | LFIA IgG | 0.0202 |
| ELISA IgM-IgG | LFIA IgM | 0.0382 |
| LFIA IgM-IgG | LFIA IgG | 0.0341 |

*Only tests comparisons with sensitivity p < 0.05 shown

Table S4: Comparison of overall sensitivity and specificity

| **Classification 1** | **Classification 2** | ***Overall sensitivity (p value)** | **Overall specificity**  **(p value)** |
| --- | --- | --- | --- |
| CLIA IgG | LFIA IgM | 0 | 0.1852 |
| CLIA IgM-IgG | CLIA IgG | 0.0195 | 0.7754 |
| CLIA IgM-IgG | LFIA IgM | 0.0066 | 0.7484 |
| CLIA Total antibody | LFIA IgM | 0.0034 | 0.8292 |
| ELISA IgG | LFIA IgM | 0 | 0.0713 |
| ELISA IgM-IgG | CLIA IgG | 0.0204 | 0.9071 |
| ELISA IgM-IgG | LFIA IgM | 0 | 0.6606 |
| LFIA IgM-IgG | LFIA IgM | 0.0367 | 0.1148 |

*Only tests comparisons with overall sensitivity p < 0.05 shown

Table S5: Comparison of overall sensitivity, specificity as per the test antigen

| **Classification 1** | **Classification 2** | ***Overall sensitivity**  **(p value)** | **Overall specificity**  **(p value)** |
| --- | --- | --- | --- |
| CLIA IgG N | LFIA IgG N | 0.0141 | 0.6475 |
| CLIA Total antibody RBD | CLIA IgG N | 0.0021 | 0.4192 |
| CLIA Total antibody RBD | ELISA IgG N | 0.0223 | 0.0755 |
| CLIA Total antibody RBD | ELISA IgG RBD | 0.186 | 0.0336 |
| ELISA IgM-IgG N | CLIA IgG N | 0.0492 | 0.8512 |
| ELISA IgM-IgG N | ELISA IgG N | 0.0074 | 0.1357 |

N, nucleocapsid; RBD, receptor-binding domain. *Only tests comparisons with overall sensitivity p < 0.05 shown

Table S6: Summary of specificity and sensitivity 7 days post symptom onset based on different antigens for CLIA, LFIA and ELISA

| **Name of the serologic test** | **Test method** | **Test antigen** | **Sensitivity 7 days post symptom onset n (%)** | | | | | | **Reference** |
| --- | --- | --- | --- | --- | --- | --- | --- | --- | --- |
|  |  |  | **IgA** | **IgM** | **IgG** | **IgM+IgG** | **IgA+IgM** | **Total Ab** |  |
| Elecsys® (Roche Diagnostics, Basel, Switzerland) | CLIA | N |  |  |  |  |  | 34 (2.9) | 35 |
| EDI™ assay (Epitope Diagnostics Inc. San Diego, CA) | ELISA | N |  | 34 (5.9) | 34 (2.9) | 34 (8.8) |  |  | 35 |
| (Lizhu, Zhuhai, China) | ELISA | N |  | 22 (31.8) | 22 (31.8) | 22 (40.9) |  |  | 52 |
| Abbott Laboratories, IL,USA | CLIA | N |  |  | 125 (53.1) |  |  |  | 20 |
| Elecsys®(Roche Diagnostics, Basel, Switzerland) | CLIA | N |  |  |  |  |  | 61(39) | 24 |
| Abbott Laboratories, IL,USA | CLIA | N |  |  | 61 (26) |  |  |  | 24 |
| Abbott Laboratories, IL,USA | CLIA | N |  |  | 81 (8.6) |  |  |  | 24 |
| EDITM (Epitope Diagnostics Inc., San Diego, CA) | ELISA | N |  |  | 38 (2.6) |  |  |  | 70 |
| Abbott Laboratories, IL,USA | CLIA | N |  |  | 38 (0) |  |  |  | 70 |
| Abbott Laboratories, IL,USA | CLIA | N |  |  | 20 (20) |  |  |  | 71 |
| Elecsys® (Roche Diagnostics, Indianapolis, IN) | CLIA | N |  |  |  |  |  | 20 (20) | 71 |
| EDITM (Epitope Diagnostics Inc.,San Diego, CA | ELISA | N |  | 30 (17) | 30 (50) | 30 (53) |  |  | 73 |
| ARCHITECT i2000SR (Abbott Laboratories, Chicago, USA) | CLIA | N |  |  | 80 (10) |  |  |  | 69 |
| Cobas e411 (Roche Diagnostics GmbH, Mannheim, Germany) | ECLIA | N |  |  |  |  |  | 80 (8.8) | 69 |
| COVID-PRESTO® (AAZ-LMB) | LFIA | N |  |  |  | 20 (10) |  |  | 65 |
| COVID-DUO® (AAZ-LMB) | LFIA | N |  |  |  | 14 (35.71) |  |  | 65 |
| Dynamiker Biotechnology [Tianjin]Co., Ltd., China | LFIA | N |  |  |  | 61 (30) |  |  | 24 |
| COVID19SEROSpeed IgM/IgG (BioS) | LFIA | N |  |  |  | 68 (69.1) |  |  | 64 |
| SD biosensor, Chungcheongbuk-do, Republic of Korea | LFIA | N |  | 16 (37.5) | 16 (43.47) | 16 (81.25) |  |  | 22 |
| Servibio® COVID-19 Sign IgM/IgG | LFIA | N |  | 30 (23) | 30 (30) | 30 (40) |  |  | 73 |
| Elecsys Anti-SARS-CoV-2 assay (Roche) | CLIA | N |  |  |  | 20 (15) |  |  | 39 |
| Abbott Alinity SARS-CoV-2 IgG assay | CLIA | N |  |  | 13 (38.46) |  |  |  | 38 |
| Roche Total antibody | CLIA | N |  |  |  |  |  | 13 (46.15) | 38 |
| Abbott Ireland DiagnosDiagnostics Division, Sligo, Ireland | CLIA | N |  |  |  | 16 (50) |  |  | 22 |
| Abbott Ireland DiagnosDiagnostics Division, Sligo, Ireland | CLIA | N |  |  | 16 (43.75) |  |  |  | 22 |
| Abbott Architect i4000SR | CLIA | N |  |  | 81 (8.6) |  |  |  | 25 |
| Hotgen, Beijing, China | ELISA | S |  | 22 (36.4) | 22 (40.9) | 22 (45.5) |  |  | 52 |
| Ortho-Clinical Diagnostics Vitros (Rochester, NY) | CLIA | S |  |  | 38 (2.6) |  |  |  | 70 |
| Siemens Healthcare Diagnostics Inc. (Tarrytown, USA) | CLIA | S |  |  |  |  |  | 13 (76.9) | 49 |
| Schweitzer Biotech Company (Taipei, Taiwan) | ELISA | S |  |  |  | 13 (61.5) |  |  | 49 |
| Wondfo (Guangzhou Wondfo Biotech Co., Ltd., China) | LFIA | S |  |  |  |  |  | 61 (39) | 24 |
| ASK Rapid Test (TONYAR Biotech  Inc. Taiwan) | LFIA | S |  |  |  | 61 (39) |  |  | 24 |
| BIOSYNEX COVID-19 BSS | LFIA | S |  | 30 (30) | 30 (7) | 30 (30) |  |  | 73 |
| Euroimmun AG, Luebeck, Germany | ELISA | S1 |  |  | 38 (10.5) |  |  |  | 70 |
| Thermo Fisher Scientific, Inc. (MA, USA) | FEIA | S1 |  |  |  |  |  | 13 (53.9) | 49 |
| Atellica IM SARS-CoV-2 assay (Siemens) | CLIA | S1 |  |  |  | 20 (15) |  |  | 39 |
| Anti-SARS-CoV-2 ELISA (IgG) (Euroimmun) | ELISA | S1 |  |  | 16 (37.5) |  |  |  | 22 |
| Anti-SARS-CoV-2 ELISA (IgA) (Euroimmun) | ELISA | S1 | 16 (37.5) |  |  |  |  |  | 22 |
| Euroimmun® | ELISA | S1 | 30 (23) |  | 30 (10) |  | IgG+IgA 30 (23) |  | 73 |
| QuantiVirus™ Anti-SARS-CoV-2 IgG Test | Flow cytometry | S1 |  |  | 13 (46.15) |  |  |  | 33 |
| LIAISON (DiaSorin S.p.A., Saluggia, Italy) | CLIA | S1 & S2 |  |  | 20 (10) |  |  |  | 71 |
| LIAISON SARS-CoV-2 assay (DiaSorin) | CLIA | S1/S2 |  |  | 20 (20) |  |  |  | 39 |
| In house | CLIA | S & N |  |  | 63 (80.95) |  |  |  | 66 |
| In house | CLIA | S & N |  | 63 (82.54) |  |  |  |  | 66 |
| Beckman Coulter Diagnostics, Inc. (Brea, USA) | CLIA | RBD |  |  |  | 13 (61.5) |  |  | 49 |
| Abbott Architect platform (Abbott Park, IL, USA) | CLIA | N/RBD |  | 132 (24.6) | 138 (23.2) |  |  |  | 54 |
| Vircell Microbiologists- Spain | ELISA | Not specified |  |  |  |  |  | 25 (76) | 29 |
| Vircell Microbiologists- Spain | ELISA | Not specified |  |  | 25 (36) |  |  |  | 29 |
| Allserum EIA COVID-19 IgM (Mbiolog Diagnosticos Ltda. - Brazil) | ELISA | Not specified |  | 24 (16.7) |  |  |  |  | 29 |
| Allserum EIA COVID-19 IgG (Mbiolog Diagnosticos Ltda. - Brazil) | ELISA | Not specified |  |  | 24 (20.8) |  |  |  | 29 |
| Euroimmun AG, Luebeck, Germany | ELISA | Not specified | 15 (33.3) |  |  |  |  |  | 29 |
| Euroimmun AG, Luebeck, Germany | ELISA | Not specified |  |  | 15 (6.7) |  |  |  | 29 |
| Xiamen InnoDx Biotech Co., Ltd. Xiamen, China) | CLIA | RBD |  | 26 (34.6) |  |  |  | 26 (53.8) | 51 |
| One Step COVID-2019 Test (Guangzhou Wondfo Biotech CO., LTD. -China) | LFIA | Not specified |  |  |  | 25  (24.0) |  |  | 29 |
| Fundação Oswaldo, Cruz - Brazil | LFIA | Not specified |  | 25 (28) | 25 (24) | 25 (40) |  |  | 29 |
| ECO Teste (Eco Diagnostica Ltda -  Brazil) | LFIA | Not specified |  | 25 (28) | 25 (20) | 25 (28) |  |  | 29 |
| Qingdao Hightop, Biotech CO., LTD. -  China | LFIA | Not specified |  | 25 (8) | 25 (8) | 25 (12) |  |  | 29 |
| Wama Produtos Para Laboratorio LTDA -  Brazil | LFIA | Not specified |  | 25 (20) | 25 (24) | 25 (32) |  |  | 29 |
| Gold Analisa Diagnóstica LTDA | LFIA | Not specified |  | 25 (0) | 25 (16) | 25 (16) |  |  | 29 |
| BTNX Inc Kit 1 | LFIA | Not specified |  |  | 154 (16) |  |  |  | 56 |
| BTNX Inc Kit 2 | LFIA | Not specified |  |  | 154 (13) |  |  |  | 56 |

N, nucleocapsid; S, spike glycoprotein; S1, subunit 1 of the spike glycoprotein; S2, subunit 2 of the spike glycoprotein; RBD, receptor-binding domain.

Table S7: Serological test summary of overall specificity and sensitivity based on LFIA, CLIA and ELISA

| **Name of the serologic test** | **Test method** | **Test antigen** | **Overall Sensitivity n (%)** | | | | | | **Specificity n (%)** | **Reference** |
| --- | --- | --- | --- | --- | --- | --- | --- | --- | --- | --- |
|  |  |  | **IgA** | **IgM** | **IgG** | **IgM+IgG** | **IgA+IgM** | **Total Ab** |  |  |
| (Lizhu, Zhuhai, China) | ELISA | N |  | 214 (68.2) |  |  |  |  | 100 (100) | 52 |
| (Lizhu, Zhuhai, China) | ELISA | N |  |  | 214 (70.1) |  |  |  | 100 (100) | 52 |
| (Lizhu, Zhuhai, China) | ELISA | N |  |  |  | 214 (80.4) |  |  | 100 (100) | 52 |
| (Abbott Laboratories, IL,USA) | CLIA | N |  |  | 97 (97.9) |  |  |  | 847 (99.6) | 55 |
| Abbott Laboratories, IL,USA | CLIA | N |  |  | 128 (70) |  |  |  | 62 (98.4) | 71 |
| Elecsys® (Roche Diagnostics, Indianapolis, IN) | CLIA | N |  |  | 128 (70) |  |  |  | 64 (96.9) | 71 |
| In-house | ELISA | N |  |  | 51 (65) |  |  |  | 491 (95) | 37 |
| ARCHITECT i2000SR | CLIA | N |  |  | 594 (64.48) |  |  |  | 100 (99) | 61 |
| Cobas e411 | ECLIA | N |  |  |  |  |  | 594 (80.48) | 100 (99) | 61 |
| ARCHITECT i2000SR | CLIA | N |  |  | 82 (49.4) |  |  |  | 223 (93.3) | 59 |
| ARCHITECT i2000SR | CLIA | N |  |  | 170 (38.8) |  |  |  | 163 (100) | 69 |
| Cobas e411 | ECLIA | N |  |  |  |  |  | 170 (40.6) | 163 (100) | 69 |
| COBAS 6000 | ECLIA | N |  |  |  |  |  | 145 (96) | 1193 (99.91) | 75 |
| ARCHITECT i2000SR | CMIA | N |  |  | 145 (93) |  |  |  | 1193 (99.5) | 75 |
| Architect (Abbot | CMIA | N |  |  | 104 (75.0) |  |  |  | 115 (100) | 42 |
| EDI (Epitope Diagnostics) | ELISA | N |  |  | 220 (71.8) |  |  |  | 278 (68.7) | 42 |
| Elecsys® (Roche Diagnostics Basel, Switzerland) | CLIA | N |  |  | 223 (71.8) |  |  |  | 113 (100) | 72 |
| Abbott Laboratories, IL,USA | CLIA | N |  |  | 223 (70.9) |  |  |  | 113 (99.1) | 72 |
| Euroimmun AG, Luebeck, Germany | ELISA | N |  |  | 223 (73.1) |  |  |  | 113 (94.7) | 72 |
| Mikrogen | ELISA | N |  |  | 223 (70.4) |  |  |  | 113 (96.5) | 72 |
| Epitope Diagnostics, Inc. (EDI), San Diego, CA | ELISA | N |  |  | 146 (78.8) |  |  |  | 578 (87) | 62 |
| ImmunoDiagnostics Limited, Sha Tin, Hong Kong | ELISA | N |  |  | 140 (76.4) |  |  |  | 306 (98.7) | 62 |
| Abbott-Architect SARS-CoV-2 assay | CMIA | N |  |  | 146 (92.5) |  |  |  | 498 (99.6) | 62 |
| Elecsys Anti-SARS-CoV-2 assay (Roche) | ECLIA | N |  |  |  | 214 (93.9) |  |  | 498 (99.6) | 62 |
| Abbott SARS-CoV-2 IgG (Abbott) | CLIA | N |  |  | 110 (91.8) |  |  |  |  | 64 |
| COVID19SEROSpeed IgM/IgG (BioS) | LFIA | N |  |  | 564 (86) |  |  |  | 215 (98.1) | 64 |
| LFIA (Technogenetics S.r.l.,Milan, Italy) | LFIA | N |  |  | 206 (84) |  |  |  | 130 (99.2) | 26 |
| LFIA (Technogenetics S.r.l.,Milan, Italy) | LFIA | N |  | 207 (47.6) |  |  |  |  | 130 (98.5) | 26 |
| Mikrogen assay IgG | ELISA | N |  |  | 105 (75.2) |  |  |  | 102 (93.1) | 30 |
| Elecsys Anti-SARS-CoV-2 assay (Roche) | CLIA | N |  |  |  |  |  | 105 (76.1) | 102 (100) | 30 |
| In-house | LFIA | N |  |  |  | 217 (95.85) |  |  | 158 (97.47) | 53 |
| Lizhu, Zhuhai, China | ELISA | N |  |  |  | 127 (83) |  |  | 70 (94.3) | 50 |
| VivaDiag (VivaChek Inc., China) | LFIA | Unspecified |  | 114 (47.4) |  |  |  |  | 198 (99.0) | 68 |
| VivaDiag (VivaChek Inc., China) | LFIA | Unspecified |  |  | 114 (46.5) |  |  |  | 198 (100) | 68 |
| Acro Biotech, Inc.,Rancho Cucamonga, CA, USA | LFIA | Unspecified |  |  |  | 73 (37) |  |  | 413 (94) | 60 |
| In-house | ELISA | S |  |  |  |  |  | 226 (94.7) | 426 (98.4) | 27 |
| Inhouse | ELISA | S |  | 150 (100) |  |  |  |  | 150 (96) | 76 |
| Inhouse | ELISA | S |  |  | 150 (99.3) |  |  |  | 150 (100) | 73 |
| SARS-CoV-2 IgG S-ELISA (in-house Region Västerbotten (in-house RV)) | ELISA | S |  |  | 278 (99.3) |  |  |  | 278 (99.3) | 42 |
| Inhouse | ELISA | S |  |  | 51 (69) |  |  |  | 100 (95) | 37 |
| Inhouse | Flow cytometry | S |  |  |  | 29 (72) |  |  | 134 (99) | 37 |
| Xiamen InnoDx Biotech Co., Ltd. Xiamen, China) | CLIA | RBD |  | 206 (72.3) |  |  |  |  | 270 (99.3) | 51 |
| Xiamen InnoDx Biotech Co., Ltd. Xiamen, China) | CLIA | RBD |  |  |  |  |  | 206 (90.8) | 270 (98.9) | 51 |
| Hotgen, Beijing, China | ELISA | RBD |  |  | 214 (74.3) |  |  |  | 100 (100) | 52 |
| Hotgen, Beijing, China | ELISA | RBD |  | 214 (77.1) |  |  |  |  | 100 (100) | 52 |
| Hotgen, Beijing, China | ELISA | RBD |  |  |  | 214 (82.2) |  |  | 100 (100) | 52 |
| Xiamen Wantai Kairui Biological Technology Co., Ltd, China) | CLIA | RBD |  |  |  |  |  | 141 (95.7) | 234 (98.7) | 74 |
| Wantai SARS-CoV-2 Ab test (Wantai) | ELISA | RBD |  |  |  |  |  | 65 (95.4) | Not assessed | 64 |
| In-house | LFIA | RBD |  |  |  | 379 (88.6) |  |  | 128 (90.63) | 46 |
| In-house | LFIA | RBD&N |  |  |  | 170 (92.9) |  |  | 300 (98.7) | 48 |
| In-house | ELISA | RBD |  |  | 379 (84.7) |  |  |  | 184 (100) | 23 |
| CLIA (Shenzhen YHLO Biotech Co., Ltd., China) | CLIA | N&S |  | 207 (54.6) |  |  |  |  | 130 (96.2) | 26 |
| CLIA (Shenzhen YHLO Biotech Co., Ltd., China) | CLIA | N&S |  |  | 207 (89.9) |  |  |  | 130 (98.5) | 26 |
| Inhouse | CLIA | N&S |  | 513 (96.69) |  |  |  |  | 972 (97.43) | 66 |
| Inhouse | CLIA | N&S |  |  | 513 (85.96) |  |  |  | 972 (97.33) | 66 |
| MAGLUMI (Snibe, China) | CLIA | N&S |  |  | 223 (68.6) |  |  |  | 113 (96.5) | 72 |
| iFlash 1800 (Shenzhen Yhlo Biotech Co. Ltd., Shenzhen, China) | CLIA | N&S |  |  | 594 (76.94) |  |  |  | 100 (100) | 61 |
| In-house | Microarray | S1 |  |  | 729 (97) |  |  |  | 542 (99.6) | 47 |
| Euroimmun AG, Luebeck, Germany | ELISA | S1 |  |  | 729 (97) |  |  |  | 542 (99.6) | 70 |
| Anti-SARS-CoV-2 ELISA (IgG) (Euroimmun, Lübeck, Germany) | ELISA | S1 |  |  | 220 (71.8) |  |  |  | 278 (99.3) | 42 |
| Anti-SARS-CoV-2 IgA ELISA assay (Euroimmun AG Luebeck, Germany) | ELISA | S1 | 185 (84.3) |  |  |  |  |  | 109 (81.7) | 26 |
| In-house immunochromatography | CLIA | S1 |  |  |  | 733 (89.22) |  |  | 223 (96.86) | 40 |
| EUROIMMUN assay IgA | ELISA | S1 | 148 (74.3) |  |  |  |  |  | 152 (93.4) | 30 |
| EUROIMMUN assay IgG | ELISA | S1 |  |  | 148 (81.1) |  |  |  | 152 (98.9) | 30 |
| LIAISON (DiaSorin S.p.A., Saluggia, Italy) | CLIA | S1&S2 |  |  | 379 (82.6) |  |  |  | Not reported | 23 |
| Liaison XL (Diasorin, Luzern, Switzerland) | CLIA | S1&S2 |  |  | 145 (90) |  |  |  | 1193 (99.7) | 66 |
| LIAISON (DiaSorin S.p.A., Saluggia, Italy) | CLIA | S1&S2 |  |  | 223 (63.2) |  |  |  | 113 (99.1) | 63 |
| Liason SARS-CoV-2 S1/S2 (Diasorin) | CLIA | S1&S2 |  |  | 206 (81.6) |  |  |  | 130 (97.7) | 17 |
| Innovita (Tangshan) Biotechnology Co., Ltd. | LFIA | SARS-CoV-2 |  | 237 (46) | 237 (95.8) | 237 (95.8) |  |  | IgM: 206 (99.5), IgG: 206 (99) IgM/IgG: 206 (98.5) | 49 |
| In house | CLIA | Synthetic peptide |  | 276 (71.4) | 276 (57.2) | 276 (81.52) |  |  | 100 (100) | 12 |
| Medical Systems Genova, Italy | CLIA | Not specified |  | (0) | (50) |  |  |  | 1084 (99.1) | 34 |
| Zydus-Kavach | ELISA | Not specified |  |  | 379 (75.7) |  |  |  | 184 (100) | 14 |
| In-house | Immunoblot | N&S |  |  |  |  |  | 223 (81)) | 379 (93) | 25 |

N, nucleocapsid; S, spike glycoprotein; S1, subunit 1 of the spike glycoprotein; S2, subunit 2 of the spike glycoprotein; RBD, receptor-binding domain.
